# Deep Brain Stimulation of Caudal Zona Incerta and Subthalamic Nucleus in Patients with Parkinson's Disease: Effects on Diadochokinetic Rate

**DOI:** 10.4061/2011/605607

**Published:** 2011-10-09

**Authors:** Fredrik Karlsson, Elin Unger, Sofia Wahlgren, Patric Blomstedt, Jan Linder, Erik Nordh, Hamayun Zafar, Jan van Doorn

**Affiliations:** ^1^Division of Speech and Language Pathology, Department of Clinical Sciences, Umeå University, SE-90185 Umeå, Sweden; ^2^Division of Clinical Neuroscience, Department of Pharmacology and Clinical Neuroscience, Umeå University, SE-90185 Umeå, Sweden; ^3^Rehabilitation Research Chair, King Saud University, Riyadh 11433, Saudi Arabia; ^4^Division of Clinical Oral Physiology, Department of Odontology, Umeå University, SE-90185 Umeå, Sweden; ^5^Department of Rehabilitation Sciences, College of Applied Medical Sciences, King Saud University, Riyadh 11433, Saudi Arabia

## Abstract

The hypokinetic dysarthria observed in Parkinson's disease (PD) affects the range, speed, and accuracy of articulatory gestures in patients, reducing the perceived quality of speech acoustic output in continuous speech. Deep brain stimulation (DBS) of the subthalamic nucleus (STN-DBS) and of the caudal zona incerta (cZi-DBS) are current surgical treatment options for PD. This study aimed at investigating the outcome of STN-DBS (7 patients) and cZi-DBS (7 patients) in two articulatory diadochokinesis tasks (AMR and SMR) using measurements of articulation rate and quality of the plosive consonants (using the percent measurable VOT metric). The results indicate that patients receiving STN-DBS increased in articulation rate in the Stim-ON condition in the AMR task only, with no effect on production quality. Patients receiving cZi-DBS decreased in articulation rate in the Stim-ON condition and further showed a reduction in production quality. The data therefore suggest that cZi-DBS is more detrimental for extended articulatory movements than STN-DBS.

## 1. Introduction

Deep brain stimulation (DBS) in the subthalamic nucleus (STN) is an established and effective treatment for motor symptoms associated with Parkinson's disease (PD). However, effects of STN-DBS on speech motor function are varying, and minor improvements, as well as stimulation-induced deterioration, have been reported [1–3]. This is also the case regarding DBS in the nucleus ventralis intermedius (Vim) of the thalamus, which is sometimes used for parkinsonian tremor, but most often for other forms of tremor [4]. Recently, the Zona incerta (Zi) has been suggested as an alternative target to the STN and Vim in PD [5, 6]. As part of a larger study we have therefore decided to evaluate the effects of Zi-DBS on speech and to compare these with the effects of STN-DBS.

Speech impairment is a frequently observed feature of PD [7]. The hypokinetic dysarthria associated with PD involves reduction in movement range in articulatory gestures [8] and, in contrast to both ataxic and spastic dysarthria, a normal [9] or indeed accelerated voluntary articulation rate [10, 11] in simple connected speech tasks. In the limited time frame available during connected speech, the active articulator often fails to reach the target location, resulting in reduced perceptual quality in the resulting speech signal.

Frequently used experimental tasks in controlled studies of speech production proficiency involve the production of repeated syllables in a fast rate (diadochokinesis, DDK). The task is administered either in the form of repeated /pa/, /ta/, or /ka/ syllables (alternating motion rate, AMR) or the repetitive production of the full sequence /pataka/ (sequential motion rate, SMR) [12]. In the first form (AMR) the speech articulation task measures the maximum rate in articulatory movement in the jaw combined with movements in the lips or anterior or posterior parts of the tongue [12]. As such, the AMR task estimates the maximum articulation rate of syllable-sized units involving maximally extended articulatory movements in the syllable onset. 

In contrast, the SMR task involves an alternation between places of constriction in the vocal tract, placing a higher load on patients' sequencing of articulatory movements [12]. Thus, although not aimed at serving as proxies for patients' production of fluent speech [13], the two DDK tasks are well suited to investigate effects in patients' articulatory proficiency and control. 

 Specifically for patients with PD, Tjaden and Watling [13] argued that the AMR and SMR tasks provide a complementary description of articulatory proficiency in the patients compared to normal controls. For the AMR task, results have shown that PD patients may have a higher articulation rate compared to healthy controls [10, 13] demonstrating that PD patients are able to manifest continuous articulatory alternations of even higher frequency than normal controls performing the same simple task. In the more complex SMR task, however, PD patients have shown a slowed articulation rate compared to normal controls [13]. Thus, it is likely that the relationship between speech articulation ability in PD patients to that of normal controls is dependent on the demands of the articulation task and consequently that articulation rate in PD patients is best evaluated using both AMR and SMR tasks [13]. 

A high articulation rate does not, however, necessarily indicate an increased articulatory proficiency. It has been proposed that an alternative way of achieving an increase in articulation rate is by increasing the articulatory undershoot in speech gestures, a feature that has been observed in PD patients' speech [8, 14, 15]. Patients would be able to increase the number of CV alternations per second by approximating the full articulatory target (articulatory undershoot), at the expense of acoustic quality. 

The plosive group of consonant speech sounds has been identified as particularly sensitive to the effects of articulatory undershoot [16]. In order for a plosive to be perceived, a silent interval followed by an acoustic transient is a required feature of the acoustic output. In order for this acoustic output to be produced, a full closure between the active and the passive articulator is required. After a period of pressure build-up driven by the lungs, the plosive is released, creating an instantaneous drop in pressure and resulting in the acoustic transient [17]. Thus, due to its strong dependence on a full range of motor action of the active articulator and a continued full closure between the articulators during the build-up of supraglottal pressure, the hypokinetic dysarthria associated with PD is very likely to have strong negative impact on the consonant produced, possibly failing to produce a stop consonant at all. In a prevocal position, where phonation is expected to follow the release of the plosive, an interaction between subglottal and supraglottal pressure may either afford or prevent phonation to occur due to the coordination of the articulatory movements involved, placing further demands on the articulatory control of the speaker.

The acoustic measure of Voice Onset Time (VOT) has successfully been employed as a yardstick for an acceptable plosive produced in prevocalic position [18]. VOT has been defined as the distance in time between the acoustic release of the plosive and the onset of voicing of the following vowel [19]. Thus, the measure requires both the presence of a detectable acoustic transient and an onset of voicing in order to be calculated. 

VOT values have been shown to be difficult to measure reliably across raters in PD patients [20]. Possibly due to this fact, studies of VOT differences between PD patients and normal speakers have produced variable results, where PD patients were found to have a longer VOT [21], shorter VOT [22], or no significant difference [23, 24] compared to normal geriatric controls. Thus, the VOT measure itself has not provided conclusive results concerning the super- and supra-laryngeal control and coordination in PD patients due to the difficulty involved in making the acoustic measurements. However, it has been argued by Özsancak et al. [25] that a more simple judgment of whether a VOT measurement could be made or not may provide a more suitable quantification of this aspect of speech production in dysarthric patients [25]. Özsancak et al. [25] argued that the relative frequency of which VOT measurements are afforded by the produced plosives correlates with the articulatory control and precision in the patient: a positive speech outcome in the disease is argued to lead to an increase in percent measurable VOT. In addition, Özsancak et al. [25] found positive correlation between measurability of VOT and Intelligibility Scores in dysarthric patients. Thus, the measure is taken to provide a productive quantification of speech motor effects due to the progression of the disease.

DBS is an established treatment for patients with PD and has been shown to reduce cardinal symptoms of PD related to motor function and control [26, 27]. For speech motor proficiency, however, the results are more mixed. In studies including the unified Parkinson's disease rating scale (UPDRS) [28] patients have been reported to show a positive effect on speech motor scores [29, 30], a differential progression in DBS-STN effect due stimulation parameters [31], or a short-term positive effect seen in 6-month [32, 33] and 1–3-year followups [33, 34], which may disappear later in the progression of the disease [26, 33]. Negative effects of DBS-STN on speech-related UPDRS motor scores (UPDRS-III) have been found both in patients treated with unilateral left hemisphere [35, 36], as well as bilateral stimulation [2, 37, 38].

More detailed investigations have been conducted concerning the nature of speech-related effects of STN-DBS. Positive outcomes have been shown in some studies for voice parameters such as mean pitch [39, 40], pitch range [39], and various measures of voice loudness and stability [39–41]. Patients have also been shown to improve in force in articulators in isolated motor tasks [42, 43] and in the variability of laryngeal and supraglottal coordination timing [39] due to STN-DBS. Other studies have reported a significant positive effect on voice intensity and pitch variability when combined with L-dopa treatment [41], but no effect of STN-DBS alone. 

STN-DBS has however also been proposed to have strong adverse effects on speech production proficiency. Several investigations have found stimulation-induced worsening in speech articulation primarily in bilateral STN stimulation [44, 45] or for patients stimulated unilaterally in the left hemisphere [35, 36]. Thus, despite the positive effects achieved by stimulation on general motor proficiency [26], specific features of speech articulation may be worsened in patients stimulated in STN.

The STN is, however, not the only target in DBS for PD. Recently, a nonrandomized study demonstrated the caudal zona incerta (cZi) to be a more efficient target regarding UPDRS-III than the STN [5]. The cZi target was also described by Plaha et al. [5] as not having the adverse effects on speech and balance as other areas closer to the STN. The cZi might therefore be considered a promising target for DBS in terms of speech outcomes.

The aim of the present study was to investigate the articulatory proficiency in terms of articulation rate and accuracy in a syllable repetition task in PD patients treated with cZi-DBS compared to patients treated with STN-DBS.

## 2. Method

### 2.1. Patients

Fourteen consecutive patients (10 males and 4 females, aged between 49 and 72 years) with idiopathic PD were included in this prospective nonrandomized study. The patients had been selected on clinical grounds for DBS surgery. Thus they were not recruited into the current study on the basis of their speech status. The patients were operated on between 2005–2007 (STN group) and 2008-2009 (cZi group). The clinical selection criteria for the patients' suitability for surgery were the same for both groups. 

These patients also participated in an accompanying study on the comparative effects of cZi-DBS and STN-DBS on voice intensity [46].

The surgical procedures for the respective targets have been previously described in detail [4, 5]. Seven consecutive patients were implanted bilaterally (5) or unilaterally (left) (2) in the STN, followed by seven implanted bilaterally in the cZi. An overview of patients is presented in Table 1. The study has been approved by the Regional Ethical Review Board in Umeå (Dnr: 08-093M; 2008-08-18). 

### 2.2. Surgical Procedure

Targets and trajectories were identified on MRI using the Frame Link planning station (Medtronic, Minneapolis, MN, USA). In the STN the target was chosen at a line connecting the anterior borders of the red nucleuses, at the level of their maximal diameter, 1.5 mm lateral of the medial border of the STN. The target in the posterior subthalamic area (PSA) was chosen at the same level and slightly posteriorly medially to the STN [11]. The electrode implantation was performed in local anesthesia, and the effect was evaluated using macrostimulation. A stereotactic CT was performed during surgery, and the images were fused with the preoperative MRI for identification of the electrode position. 

### 2.3. Speech Samples

The speech material was selected from recordings made in three clinical conditions: at baseline before surgery where the patient was medicated with a levodopa test dose equivalent to 1.5 times their normal levodopa dosage, and then Off and On stimulation (one hour after the stimulation was switched off and on, resp.) 12 months after surgery. The postoperative recordings were made within the optimal period of the patient's normal medication cycle. 

The recordings were made in a sound-treated booth, using a calibrated head-mounted microphone (Sennheiser MKE 2 P-C), with a 15 cm mouth to microphone distance. The samples were recorded on a digital audio flash recorder (Marantz PMD 660) or in the case of some early recordings a digital audio tape recorder (Panasonic SV 3800). A calibration tone (80 dB, 1 kHz) was used at the beginning of each recording. 

The speech material used in this study consisted of a syllable repetition task. In the early recordings the patients were instructed to repeat each syllable /pa/, /ta/, and /ka/ as fast and for as long as they could. In the more recent recordings, the instructions were refined so that the patients were given more specific information with an auditory model of the task, and they first practiced by repeating the syllables evenly at normal tempo, before proceeding to their fastest possible even tempo. This refinement in instructions was made in collaboration with other research centers in Sweden to ensure comparability of speech data collected from PD patients. Five STN patients received the earlier instructions in all testing conditions, and six cZi patients received the refined instructions in all conditions. The remaining two STN and one cZi patients received the earlier instructions for the preoperative test and the refined instructions for the postoperative tests. The procedure was performed in two sequences, first using sequences of identical syllables /pa/, /ta/, and /ka/ (AMR) and then again using a basic pattern /pataka/ which was then repeated in the same way (SMR). 

### 2.4. Acoustic Analysis

All speech measurements were made from the display of the acoustic waveform presented by the Wavesurfer (version 1.8.5) software package [47]. The measurements were performed by the second and third authors in a random order in terms of patient and treatment condition in order to reduce the possibility of systematic measurement effects across patients. All syllable sequences were examined to determine their suitability for inclusion. The criterion for inclusion of a sequence was that it must have consisted of at least 6 syllables (AMR) or 4 syllables (SMR), where a syllable was defined as measurable if it consisted of an increase of energy followed by a period of silence or reduced energy in the waveform [13]. In the AMR productions, acoustic measurements of syllable duration were collected for syllable repetition 2–11 (10 syllables) or as many syllables the patient was able to produce (a minimum of 6). The first syllable was excluded because the initial silent closure phase in plosives in word-initial position makes it not comparable to medial and final plosives in terms of measurable duration. Articulation speed was then estimated by the total duration of the measured sequence divided by the number of syllables in the sequence (syllables/s). Landmarks for VOT measurements (release transient and voicing onset [19]) were also identified and their combined presence in the signals noted as VOT being measurable (both features present) or not measurable. Syllables where either a release transient or voicing onset was not present were marked as not measurable. Continuously voiced plosives were also marked as it signifies a lack of control in laryngeal functioning, resulting in a nonplosive (an approximant of voiced fricative) being produced. The percent measurable VOT metric was then calculated as the number of syllables meeting the VOT criteria, divided by the total number of syllables in the sequence, expressed as a percentage.

In the SMR material, the full /pataka/ production sequences were measured in terms of their duration, discarding the first sequence due to the effect of the utterance-initial silent phase. Up to 10 full sequences were measured if present. A minimum of 4 full sequences was set as a lower limit for inclusion of the sequence in the data set in order to ensure that each mean estimate was based on at least three points of data. Similar to the AMR productions, articulation speed was estimated by the total duration of the measured sequence divided by the number of syllables in the sequence (syllables/s). In addition, VOT landmarks were identified for the (up to 30) produced syllables in the selected production sequences and their combined presence in the signals noted as VOT being measurable or not measurable. As in the AMR data, continuously voiced plosives resulted in the plosive being judged as not measurable, as VOT is not defined for this production pattern. The percent measurable VOT metric was then calculated as for AMR.

### 2.5. Reliability

The measurements of relative frequency of measurable VOT and speech rate were repeated for 10% of the samples by two independent raters (the second and third author) in order to estimate the interrater agreement. The exact agreement in ratings of measurability of VOT was established at 93.8%  (*κ* = 0.87). Differences in estimates of speech rates were within 0.047 syllables/s in 75% of the cases, within 0.21 syllables/s in 83.3% of the cases, and within 0.69 syllables/s in 100% of the cases.

### 2.6. Statistical Analysis

Between-within (2 × 3) analyses of variance were conducted to test for statistical significance of differences in articulation rate and degree of measurable VOT related to stimulation target (STN versus cZI) and recording condition (baseline, Stim On, and Stim Off) as well as interactions between these variables. Within the data collected during the AMR speech task, effects of syllable type (/pa/, /ta/, or /ka/) were included in the analysis in a 2 × 3 × 3 ANOVA. The relationship between articulation rate and the relative frequency of measurable VOT was investigated using linear regression models.

## 3. Results

### 3.1. Articulation Rate

**Figure 1:**
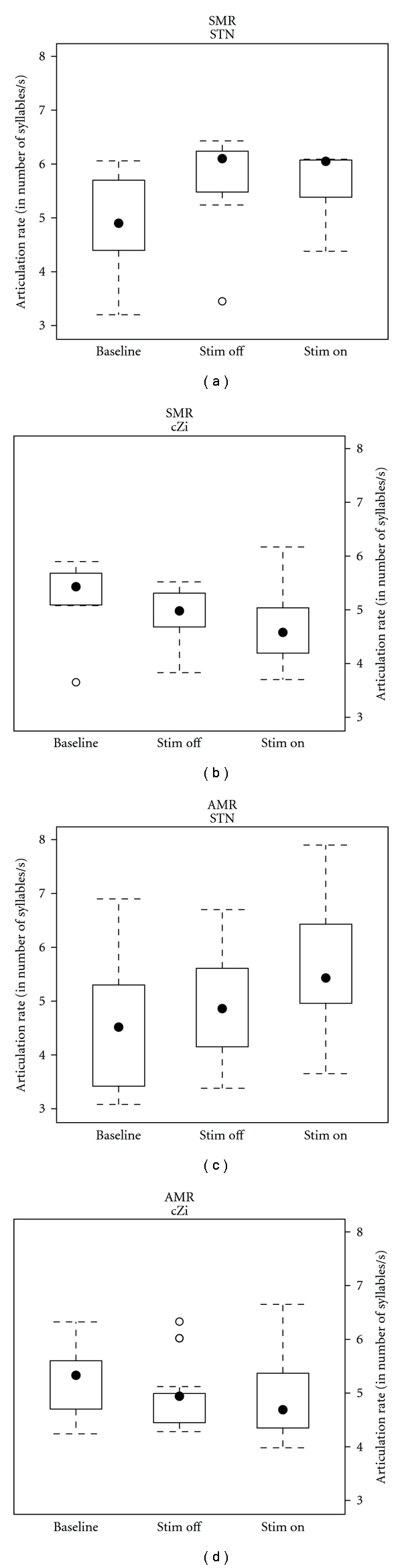
Box plot showing articulation rate under the three stimulation conditions for the two investigated patient groups and speech tasks.

The articulation rates in the AMR and SMR tasks were analyzed for treatment effects separately for cZi and STN patients On and Off stimulation compared to baseline. The results are presented in Figure 1, divided according to articulation task (AMR or SMR), stimulation condition (baseline, Stim OFF, or Stim ON), and stimulated target (STN or cZi). Within the AMR data, separate analysis was performed for the different syllable types (/pa/, /ta/, and /ka/) produced by the patients. 

For the STN patients, mean number of syllables per second increased from 5.13 to 5.54 in the AMR task, but remained at 5.63 in the SMR task. In the cZi patients, the results from the AMR task showed a decrease in number of syllables produced per second Stim ON compared to Stim OFF. In the SMR task, the number of syllables per second decreased from 4.90 in Stim OFF to 4.70 in Stim ON. In the AMR task, STN-DBS stimulation increased the mean articulation rate for all syllable types (5.05 to 5.85 for /p/, 5.06 to 5.44 for /pa/, and 4.99 to 5.32 for /ka/). The cZi-DBS group results were more mixed (4.95 to 5.25 for /p/, 4.95 to 4.72 for /pa/, and 4.87 to 4.53 for /ka/).

The articulation rates in the two tasks and three stimulation conditions were tested using a 2 × 3 × 3 ANOVA, with condition and task interaction included. The results showed a significant main effect of stimulation condition (*F*_(2,96)_ = 5.98, *P* = 0.003) and of stimulated target (*F*_(2,96)_ = 5.35,* P* = 0.02), but no significant main effect or interaction effect involving syllable type. A Tukey “Honest Significant Differences” post hoc test confirmed the significant increase in articulation rate in the AMR task due to STN stimulation (*F*_(2,96)_ = 5.84, *P* = 0.004) and the overall (task independent) reduced articulation rate in cZi compared to STN Stim ON (*F*_(2,96)_ = 5.84, *P* = 0.024) but no other investigated contrast.

A separate analysis into articulation rate effect in the bilaterally and unilaterally operated STN-DBS patients showed no systematic effect of laterality. The two patients treated with unilateral (left) stimulation showed tentative signs of opposite treatment effects. Further, for each of these observed effects, a similar effect in both size and direction could be observed in at least one bilateral STN-DBS patient. Thus, no support for a differing effect of bilateral and unilateral (left) STN in terms of articulation rate was provided by the present data.

### 3.2. Percent Measurable VOT

**Figure 2:**
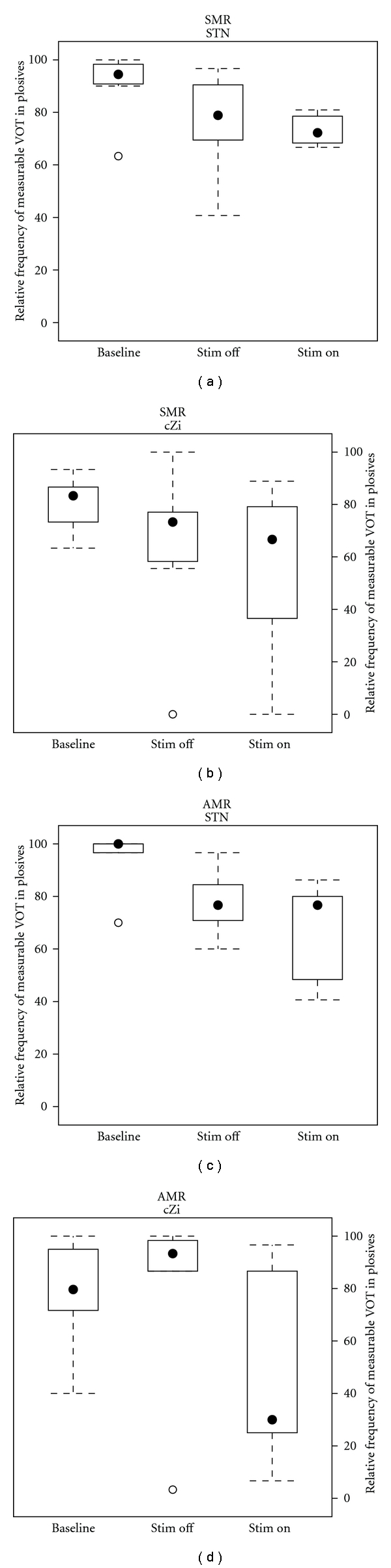
Relative frequency of measurable VOT in plosives produced under the three stimulation conditions for the two investigated patient groups and speech tasks.

The quality of articulation was estimated using the relative frequency of measurable VOT metric, and the results are presented in Figure 2. For the STN patients performing the AMR task, mean rate of measurable VOT was 77.7% in Stim OFF and was reduced to 65.8% in Stim ON. In the SMR task, 76.6% of the syllables contained the necessary criteria for VOT measurement in Stim OFF, compared to a mean rate of 73.4% in Stim ON. 

For the cZi patients, 81.0% of the productions were measurable in the AMR task under Stim OFF, but only 51.0% in Stim ON. In the SMR task, the patients performed worse compared to the AMR task (63.5% in Stim OFF), which was then reduced to a value similar to the AMR task in Stim ON (55.3%). 

Statistical testing using a 2 × 3 × 3 ANOVA with articulation task, stimulation condition, stimulated target, and syllable type (including interactions) showed a significant main effect of stimulation condition (*F*_(2,72)_ = 7.9, *P* < 0.001) only. A Tukey “Honest Significant Differences” post hoc test confirmed the significant reduction in rate of measurable VOT in cZi patients (*F*_(2,72)_ = 3.72, *P* = 0.037), but no other investigated contrasts.

A separate analysis of differences between the bilaterally and unilaterally operated STN-DBS patients showed no systematic effect of laterality. The two patients treated with unilateral (left) stimulation showed a similar effect in both size and direction to what was observed for at least one bilateral STN-DBS patient. Thus, no support for a differing effect of bilateral and unilateral (left) STN in terms of articulation data was provided by the present data.

### 3.3. Association between Articulation Rate and Percent Measurable VOT

The association between articulation rate and articulatory precision was investigated by the correlation between the computed numbers of syllables per second produced and the relative frequency of measurable VOT values in the plosives produced using the Pearson's product-moment correlation coefficient. The results showed a significant (*t*_(82)_ = −3.57, *P* < 0.001) negative overall correlation between articulation rate and relative frequency of measurable VOT (*ρ* = −0.37). A more detailed analysis of correlations between measurements within cells created by combinations of stimulation condition (baseline, Stim ON, and Stim OFF) and stimulated target showed a significant correlation only in postsurgery conditions for cZi patients (Stim ON: *ρ* = −0.60, *t*_(12)_ = −2.59, *P* = 0.023; Stim OFF: *ρ* = −0.604, *t*_(12)_ = −2.63, *P* = 0.021). No significant correlation was found for STN patients. 

The association between the two quantities was further investigated using linear regression fitted to the data within cells created by combinations of stimulation condition and target where a significant correlation had been shown. The regression line fitted to the data showed a slope of −0.010 (SE_(slope)_ = 0.0038) for Stim OFF (adjusted *R* ^2^ = 0.313) and −0.014 (SE_(slope)_ = 0.0054) for Stim ON (adjusted *R* ^2^ = 0.306). Thus, a clear impact of an increase in articulation rate on articulatory precision was observed. As the standard errors of the slope estimates overlap almost completely, the association between articulation rate and articulatory precision (as measured by relative frequency of measurable VOT) was considered equal between postsurgery conditions for the cZi patient group.

The effect of the refinement of instructions given to the patients was evaluated using Welch two-sample *t*-tests comparing the articulation rate in patients receiving an auditory model and patients not receiving an auditory model in the baseline recordings, for each task and repeated syllable separately. The results showed no effect of the auditory model (*t*_(11.05)_ = −1.4373, *P* = 0.18 for /pa/, *t*_(11.77)_ = −1.7843, *P* = 0.10 for /ta/, *t*_(11.87)_ = −0.9014, *P* = 0.38 for /k/, and *t*_(8.08)_ = −0.6301, *P* = 0.54 for /pataka/) between the groups. Further, no indication of an effect of instruction modification was perceived within the treatment groups: patients receiving the other set of instructions (earlier or refined depending on treatment group) did not make up extremes within the groups, and for each of these patients a comparable patient in terms of mean and variation in articulation rates could be observed in the data set. Thus, the results reported here did not show evidence of a systematic effect of the refinement in instructions given for the tasks.

## 4. Discussion

The present aim was to investigate and compare the speech production rate and accuracy in patients treated with STN and cZi DBS, as quantified by mean number of syllables per second and relative frequency of measurable VOT in plosive consonants [25]. Two speech tasks were investigated, the AMR task and the more complex SMR task.

The results indicate an increase in articulation rate in STN patients performing the AMR task in the stimulated condition and a decrease in articulation rate in cZi in the same condition, regardless of task performed. At Off stimulation the patients did not perform significantly differently compared to the presurgery baseline. Thus, the results indicate a reduction in articulation rate due to cZi stimulation and an (task dependent) increase in rate of articulation due to STN stimulation.

In terms of articulatory precision, the STN group appears not to be significantly affected by stimulation. No significant differences were obtained between conditions or between tasks for this group. For the cZi group, however, a significant decrease in relative frequency of measurable VOT was observed in Stim ON. Thus, with stimulation turned on, cZi patients showed signs of a significant increase in articulatory undershoot [48] and a reduction in the articulatory control needed to achieve a plosive with the appropriate perceptual characteristics.

The articulatory undershoot was also shown to be partly dependent on the rate of articulation in the task, but only significantly so in specific conditions. In the postsurgery recording for cZi patients, productions were significantly reduced in articulation quality with increase in articulation rate. The linear regression applied indicated that an increase in articulation rate with one syllable per second caused, on average, a reduction in the relative frequency of measurable VOT for the cZi group of 25 or 35 points on a percentage scale. While the data to which the linear regression was fitted show a substantial variation, with a 0.306–0.31 R^2^ value for the fit, the results show a significant overall correlation between the articulation rate and the production quality which is attributed to a strong correlation between the two quantities in the cZi patients in both Stim ON and Stim OFF. 

Two of the patients in the STN-DBS were under unilateral stimulation, while the other five patients were bilateral patients. Lateralization effects of STN-DBS on various aspects of speech have been reported previously [35, 49]. Wang et al. [49] showed a significant increase in syllable rate in STN-DBS (right) compared to STN-DBS (left) patients. However, bilateral data was not provided by Wang et al. The investigation by Santens et al. [35] provided no acoustic measurements, but was based on perceptual measurements. In their results, unilateral (left) STN-DBS provided no significant differences between judgments of speech prosody, articulation, and intelligibility by trained professionals compared to bilateral stimulation. Thus, in as far as the articulation results from Santens et al. [35] are comparable to the percent measurable VOT metric presented here, inclusion of unilateral patients is unlikely to have affected the outcomes of the present study. Furthermore, we found that removing the unilateral patients from the data set still afforded the same conclusion and also for each one of the two unilateral patients, a similar treatment effect (both in size and direction) could be observed in a bilateral STN-DBS patient. We therefore feel assured that the unilateral patients do not differ significantly from the bilateral patients in (supraglottal) articulatory control, which seems to be in agreement with the Santens et al. data [35]. 

The measurements of measurability of VOT and articulation rate were derived from the acoustic signal and were conducted in a randomized procedure, but the experimenters were blinded only to the treatment localization of the patient and not to the stimulation condition. The lack of blinding of the experimenters has the potential of being a confounding factor in the results. However, the data presented here were obtained directly from the physical properties of the acoustic signal using specific criteria, and the measurements were repeated with high level of interrater reliability. Thus, the risk of the blinding factor influencing the results is highly reduced due to the nature of the data. Further, the results presented here involve interactions between the task performed, stimulation condition (to which raters were not blinded) and targeted localization (to which the raters were blinded). Further, no comparable speech data had been made available in the literature for cZi patients at the time the measurements were made. With a randomized procedure, experimenters being blinded to at least one factor in the significant interactions and not having any information on which to base expectations, the chance of the lack of blinding being a confounding factor in the results presented here is judged to be very small. Thus, we concluded that the blinding of experimenters was not a confounding factor in our results. 

The instructions given to the patients were refined in later recordings to include a practice run and an auditory model. This refinement was made to ensure comparability of our data with data collected from PD patients in other centers across Sweden. The impact of this potential confounding factor was investigated for statistical differences in articulation rate between patients receiving the two versions of instructions. The results showed no evidence of a significant effect of instructions in on the articulation rate in the speech task, and no evidence was found for patients deviating from the treatment group trend due to the instructions received. Thus, it is concluded that the change in instructions given to the patients was not a confounding factor in the present data.

Our results suggest a differentiated treatment effect of STN and cZi stimulation in terms of articulatory proficiency based on acoustic measurements. STN patients increased in articulation rate (in the simple AMR task) ON stimulation, while cZi patients decreased in articulation rate in both tasks in the same condition. Further, the quality of production decreased with cZi stimulation, but showed a much smaller (and not significant) effect of stimulation in the STN. 

These findings are in accordance with previous findings. Dysarthria is common in PD, and STN-DBS has previously been reported to improve certain aspects of speech in some patients [39–43]. Further, dysarthria in PD in positron emission tomography (PET) studies has been linked to an overactivation in the SMA and DLPFC and an underactivation in the cerebellum and primary motor cortex. A normalization of these changes by STN-DBS has been demonstrated in one study in patients where STN-DBS improved speech [45, 50]. Concerning deterioration of speech following Zi-DBS, it has recently been demonstrated that dysarthria in STN-DBS is more likely to be caused by electrodes placed more medially in proximity to the anterior Zi [2, 51], and dysarthria constituted a problem in patients implanted in the anterior Zi [5]. This side effect is probably caused by an affection of the cerebellothalamic fibers in the area. Affection of these fibers, passing into the Vim, is probably also responsible for many cases of dysarthria following Vim-DBS [51], which previously has been attributed to a spread of current to the internal capsule [52].

The acoustic results presented here have been interpreted in terms of articulatory proficiency. The quantification of articulatory proficiency (measurability of VOT) was chosen primarily because it involves a complex coordination of articulatory gestures in the glottal and supraglottal structures, and it has been used in previous research for dysarthric speakers [25]. Additionally, DDK results and measurability of VOT in particular have all been linked individually to intelligibility of speech [25, 35, 53]. Furthermore, acoustic measures are capable of detecting subperceptual speech changes and are not subject to influences of auditory perceptual bias [54]. The results from the current study provide evidence of a direct effect of STN-DBS or cZi-DBS on the patients' articulatory proficiency. As such, they indicate advances and worsening in motor control and proficiency in PD patients due to DBS-STN or DBS-cZi. 

However, it is difficult to evaluate the present results in terms of a communicative setting (such as intelligibility or comprehensibility of the speech produced). Perceived articulatory precision in patients with PD, DDK results, and measurability of VOT in particular have all been linked individually to intelligibility of speech [25, 35, 53], but it is also possible that the changes reported here are below the perceptual threshold. It is beyond the aim and scope of this paper to investigate perceptual effects of observed changes in the motor proficiency in the listener. The perceptual impact of the treatment effects found here should be targeted by further research using suitable speech material and procedures that specifically address these issues.

## 5. Conclusion

This paper has provided evidence of a DBS-induced increase in articulation rate in the AMR task for STN patients, with no significant negative effect on production quality, but a decrease in both articulate and production quality across both tasks for cZi patients. A related study [46] that used essentially the same patient groups has also shown a differential response between cZi- and STN-DBS for voice intensity. Thus, it is concluded that cZi stimulation might be more detrimental for articulatory proficiency in patients compared to stimulation of the STN. Our results must, however, be interpreted with caution, as they are based on a limited number of patients.

## Figures and Tables

**Table 1:** Characteristics of patients in the two surgical groups. Mean age as well as median unified Parkinson's disease rating scale smotor scores, UPDRS-III, (with standard deviations) are provided. There were no statistical differences between the groups for age, duration since diagnosis, or any of the UPDRS-III scores.

Characteristic	STN group (*n* = 7)	cZI group (*n* = 7)
Age (y)	62.2 ± 8.2 (51–72)	61.9 ± 9.0 (49–71)
Gender	5 M, 2 F	5 M, 2 F
Electrode placement	5 bilatera l,2 unilateral (left side)	7 bilateral
Duration since diagnosis	6.4 ± 1.5 (4–8)	5.6 ± 2.5 (2–10)
UPDRS III Off medication	39.0 (32–57)	31.0 (29–50)
UPDRS-III On medication	18.0 (6–36)	16.0 (10–42)
Speech* (UPDRS III Item 18) Off med	1.0 (0–2)	1.0 (0–2)
Speech (UPDRS III Item 18) On med	0.0 (0-1)	0.7 ± 0.5 (0–1)
